# Anatomic and radiological correlation of injectate spread from first thoracic costotransverse junction in cervical erector spinae plane

**DOI:** 10.4322/acr.2021.275

**Published:** 2021-05-06

**Authors:** Sandeep Diwan, Xavier Sala Blanch, Abhijit Nair, Dipal Shah

**Affiliations:** 1 Sancheti Hospital, Department of Anaesthesiology, Pune, Maharashtra, India; 2 Hospital Clinic University of Barcelona, Department of Anesthesia and Anatomy, Barcelona, Spain; 3 Ibra Hospital, Department of Anaesthesiology, North Sharqiya Governorate, Ibra, Sultanate of Oman; 4 Sancheti Institute of Orthopaedic and Rehabilitation, Department of Radiodiagnosis, Pune-Maharashtra, India

**Keywords:** Anesthesia conduction, Ultrasonography, Nerve block, Dissection, Cadaver

## Abstract

**Introduction:**

Cervical erector spinae plane block (ESPB) provides postoperative pain relief when administered at the level of first thoracic costotransverse junction (CTJ) for surgeries on the proximal shoulder and cervical spine. We propose to describe the spread of 20 ml radiocontrast - dye solution administered at this level from caudad to cephalad direction in a fresh frozen cadaveric model through imaging and cross-sections.

**Methods:**

An observational study with four thoracic to cervical ESP blocks at the level of first thoracic CTJ level on two fresh cadavers (total 4 specimens) was conducted using 20 ml of radiocontrast- methylene blue combination (10 ml through the needle and 10 ml through the catheter). Both cadavers were subjected to computed tomography (CT) scan. An anatomist and radiologist, respectively, analyzed cross-sections of cadavers and CT contrasted images.

**Results:**

The spread was assessed in axial, sagittal, and coronal at the levels of C4, C5, C6, C7and T1. The medial limit was articular processes in both cadavers. The lateral limits were the outer border of the middle scalene muscle in cadaver 1 and posterior to the sternocleidomastoid muscle in cadaver 2. Contrast spread was visualized on the superior and anterior aspect of anterior scalene muscle in cadaver 2. An epidural spread was observed at the level of C5-6 and C6-7 in axial and coronal planes in cadaver 1.

**Conclusions:**

The cervical ESPB administered at the first thoracic CTJ with injections directed cephalad has a consistent action on the dorsal spinal nerves of thoracic and cervical area, and spreads in the paravertebral space dorsal to the ventral cervical roots.

## INTRODUCTION

Erector spinae is a group of muscles which is located bilaterally along the length of the spine and forms the intermediate or deep layer of intrinsic muscles of the back. The three principal muscles of the erector spinae group are (i) spinalis, (ii) longissimus, and (iii) iliocostalis. At the cervical area the erector spinae muscles are spinalis capitis and cervicis, longissimus capitis and cervicis, and iliocostalis cervicis and thoracis. At the thoracic level the muscles involved are spinalis thoracis, longissimus thoracis and iliocostalis thoracis.^1^

Introduced in the clinical practice, the thoracic erector spinae plane block (ESPB) has strongly influenced the perioperative pain management as a part of multimodal analgesia in a multitude of surgical procedures such as thoracic, abdominal, and spinal surgery.^2^^-^^6^ Several case reports have described the use of cervical ESPB for forequarter amputation and shoulder arthroplasty.^7^^,^^8^ A case series describes the cervical ESPB as a phrenic sparing block with adequate postoperative analgesia for shoulder arthroscopic surgeries.^9^ Our study primarily aims to inject a combined solution of radiocontrast – methylene blue dye (MBD) at the first thoracic costotransverse junction (CTJ) with bevel directed cephalad. Subsequently, we intended to observe the spread with computed tomography (CT) scan followed by anatomical cross-sections to demonstrate the distribution pattern at the cervical and the thoracic root level to evaluate its dissemination in relation to the spinal nerves. The secondary goal was to evaluate the contrast and dye distribution in other potential planes like prevertebral fascia, fat planes beneath the sternocleidomastoid (SCM) muscle, investing layer of deep fascia over the scalene muscle, interscalene groove, and spaces such as interscalene and epidural space.

## METHODS

After the approval from the University of Barcelona’s ethical and scientific committee, an observational study was conducted on 2 fresh cadavers, bilaterally (total 4 specimens). The cadavers were devoid of any infectious medical pathologies, scoliotic deformations, or previous surgeries at the level of the cervical or thoracic spine. Prior to dissection, the cadavers were stored at minus 20 degrees, and before dissection, they were kept for 4 hours at an ambient temperature of 22-23°C.

### Description of cervical ESPB technique

**Figure 1:**
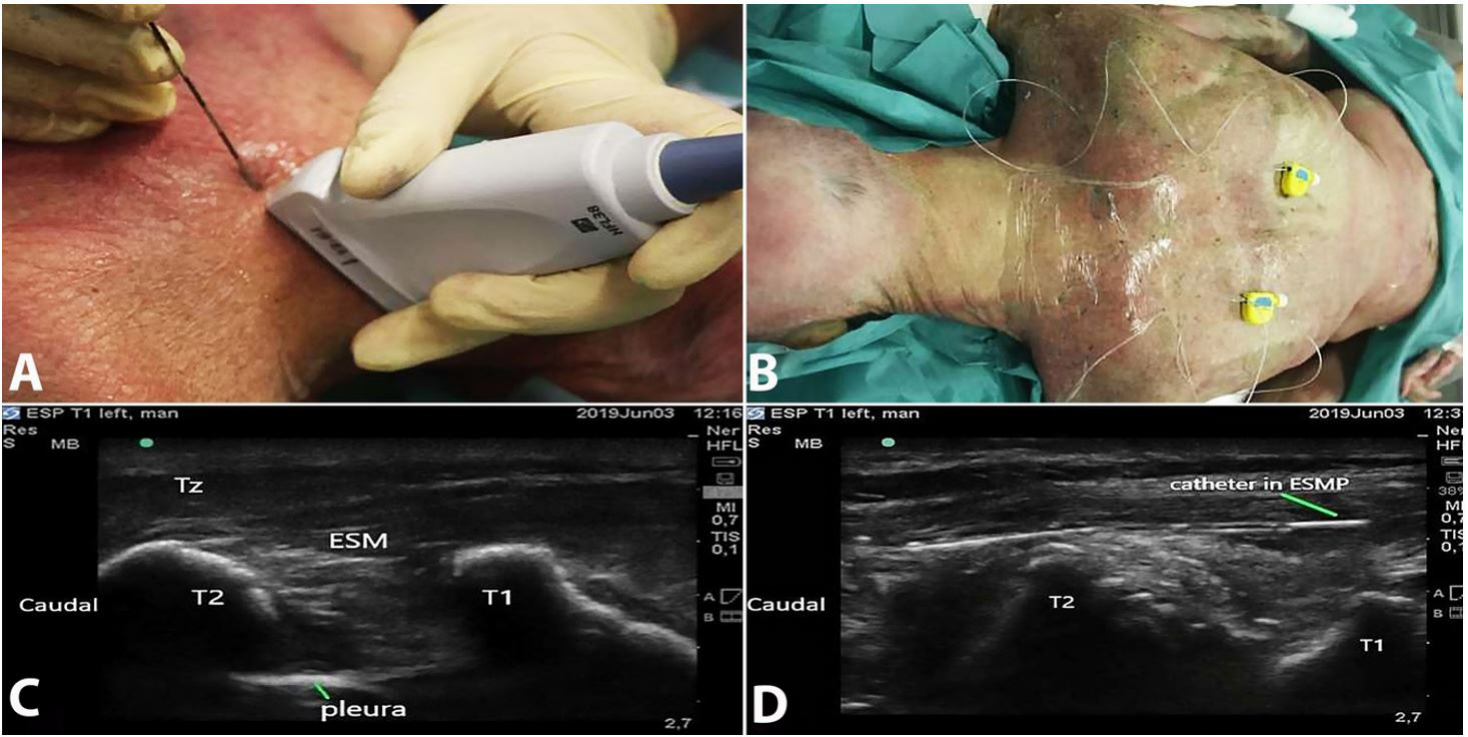
**A** – 18G Tuohy needle insertion at the first thoracic costotransverse junction; **B** – Bilateral insertion of catheters; **C** – Identification of T1-CTJ (Tz -Trapezius; ESM -erector spinae muscle); **D** – Catheter tip identified in the ESMP (erector spinae muscle plane).

The cadavers were placed in a prone position with an elevation of about 10 cm at the level of the nipples, and the head was stabilized on a headrest. The arms were extended along the body. The ultrasound (US) scan was performed with a high-frequency linear probe (5-13 mHz), (Sonosite inc., Bothell, WA, USA). The fascial plane of the upper thoracic erector spinae muscle (ESM) was located at the level of the first thoracic CTJ. The first rib was located in the supraclavicular fossa at the mid-clavicular line, following which the probe was shifted longitudinally and dorsally. Subsequently, the transducer was lateralized to identify the transverse process (TP) corresponding to T1. The probe was shifted medially to identify the first thoracic CTJ, and the target point was located dorsal to it and deep to the anterior sheath of ESM. An 18 G Tuohy needle (B. Braun, USA) was introduced starting from T2 and the bevel towards the first thoracic CTJ (Figure 1A). The needle insertion was done from caudal to cranial with the bevel facing upwards to reach the dorsal and lateral edge of TP close to the medial aspect of the first thoracic CTJ. Of the 20 ml of solution (10 ml 0.9% NaCl + 9 ml of iodinated contrast + 1 ml of methylene blue), 10 ml was injected in the fascial plane through the Tuohy needle. During injection, the distribution of the injectate was assessed by continuous US scan from caudal to cephalad in the cervical erector spinae plane (ESP). The procedure was repeated on the contralateral side. Bilateral catheters (20 G. B.Braun, USA) were inserted through the Tuohy needle and were fixed to the skin after needle removal (Figure 1B). Another 10ml of the radiocontrast-dye solution was injected through the catheter, and a cephalad limit of spread was visualized in real-time.

### CT Scan-contrast study

Both cadavers were transferred to the CT suite in a prone position. A cervical to thoracic CT scan was performed (head to T5-6). The distribution of injected volume was evaluated after a three-dimensional digital reconstruction in a computer program for Mac (OsiriX DICOM viewer, Pixelmon, Switzerland). Images obtained were analyzed by a radiologist in conjunction with an anesthesiologist who performed the blocks. A separate analysis was performed of each cadaver in axial, coronal, and sagittal planes to understand contrast spread in the following locations: epidural space, neural foramina, extraforaminal area, brachial plexus, medial most spread, lateral most spread, spread in relation to ASM and to SCM muscle.

The prone cadavers were shifted from CT suite to cadaveric laboratory and were frozen at −20 degrees C for 48-72 hrs. Both cadavers underwent axial sectional cuts at cervical to the thoracic area, starting at the level of the mandible, hyoid, cricoid, and T1 spinous process in the prone position. The cut sections were 2-2.5cm. All cut sections were placed on a wooden platform with the cranial side up and were photographed with a digital single-lens reflex camera (Canon 1000D). All specimens were positioned with its cranial side up.

## RESULTS

### US details

Cervical ESPB was performed on 2 prone cadavers successfully. The identification of the first thoracic CTJ was successful in both cadavers on both sides (Figure 1C). After injection of 10ml solution through the needle, the US images depicted a cranial spread in relation to the dorsal and medial edge of the first thoracic CTJ, deep to the anterior sheath of ESM and dorsal to the TPs. Catheters were visualized deep to the ESM. (Figure 1D).

A US scan in the prone position did not reveal the solution in the interscalene, supraclavicular, and ASM areas. Injection of 10 ml of solution through the catheter demonstrated a flow deep to the ESM spreading cephalad and was traced at the level of occiput superiorly, laterally close to the outer border of middle scalene muscle and medially close to the spinous process.

### CT contrast analysis

The CT – contrast analysis depicted a consistent caudal to the cranial distribution of injectate with respect to the medial edge of the thoracic CTJ 1. A detailed spread was analyzed and demonstrated in both cadavers (Tables 1, 2).

**Table 1:** Radio-opaque contrast spread characteristics in the cadaver 1

				Neural foramen		Extraforaminal		BP		ASM	
	Root	Nf-level	Epid	Left	Right	Left	Right	Left	Right	Left	Right
Axial	C1	--	--	--	--	--	--	--	--	--	--
	C2	C1-2	no	no	no	no	no	no	no	--	--
	C3	C2-3	no	no	no	no	no	no	no	--	--
	C4	C3-4	no	no	no	d and v -h	d and v-l	no	no	no	no
	C5	C4-5	no	no	no	d and v-h	d and v-n	no	no	no	no
	C6	C5-6	yes	Yes-l	no	yes	No	no	no	no	no
	C7	C6-7	yes	Yes-D	no	yes	No	no	no	no	no
	T1	C7-T1	no	yes -D	Yes-l	yes	Yes-l	no	no	no	no
	T2	T1-2	no	Yes-l	Yes-l	no	Yes-l	yes	no	no	no
	T3	T2-3	no	No	no	no	No	no	no	no	no
	T4	T3-4	no	No	no	no	No	no	no	--	--
	T5	T4-5	no	No	no	no	No	no	no	--	--
Coronal	C1	--	--	--	--	--	--	--	--	--	--
	C2	C1-2	no	no	no	no	No	no	no	--	--
	C3	C2-3	no	no	no	no	No	no	no	--	--
	C4	C3-4	no	no	no	yes	Yes	Yes-D	Yes-D	no	no
	C5	C4-5	no	no	no	yes	Yes	Yes-D	Yes-D	no	no
	C6	C5-6	yes	no	no	yes	No	Yes-D	No	no	no
	C7	C6-7	yes	no	no	yes	No	Yes-D	No	no	no
	T1	C7-T1	no	no	no	yes	No	Yes-D	No	no	no
	T2	T1-2	no	no	no	no	No	no	No	no	no
	T3	T2-3	no	no	no	no	No	no	No	no	no
	T4	T3-4	no	--	--	--	--	--	--	--	--
	C1	--	--	--	--	--	--	--	--	--	--
Sagittal	C1		--	--	--	--	--	--	--	--	--
	C2	C1-2	--	--	--	--	--	--	--	--	--
	C3	C2-3	--	--	no	--	no	no	No	--	--
	C4	C3-4	--	--	no	--	no	no	No	no	no
	C5	C4-5	--	--	no	--	no	no	No	no	no
	C6	C5-6	--	--	no	--	no	no	No	no	no
	C7	C6-7	--	--	yes=l	--	Yes-D	no	No	no	no
	T1	C7-T1	--	--	yes-l	--	Yes-D	no	No	no	no
	T2	T1-2	--	--	no	--	no	no	No	no	no
	T3	T2-3	--	--	no	--	no	no	No	no	no
	T4	T3-4	--	--	no	--	no	no	No	--	--
	T5	T4-5	--	--	no	--	no	--	--	--	--

**Table 2:** Radio-opaque contrast spread characteristics in the cadaver 2

				Neural foramen		Extraforaminal		BP		ASM	
	Root	Nf-level	Epid	Left	Right	Left	Right	Left	Right	Left	Right
Axial	C1		no	no	no	d and v	d and v	--	--	--	--
	C2	C1-2	air	yes-D	no	d and v	d and v	--	--	--	--
	C3	C2-3	air	yes-D	yes-l	d and v	d and v	--	--	--	--
	C4	C3-4	air	yes-D	yes -D	d and v	d and v	--	--	--	--
	C5	C4-5	air	yes-D	yes-l	d and v	d and v	--	--	no	yes
	C6	C5-6	air	yes-l	yes -D	d and v	d and v	--	--	no	yes
	C7	C6-7	air	yes-D	yes-l	d and v	d and v-f	--	--	no	yes
	T1	C7-T1	air	yes-l	no	d and v-f	d and v-f	--	--	no	--
	T2	T1-2	air	yes-D	yes	d and v-f	d and v-f	--	--	--	--
	T3	T2-3	air	No	no	no	no	--	--	--	--
	T4	T3-4	air	No	no	no	no	--	--	--	--
	T5	T4-5	--	No	no	no	no	--	--	--	--
Coronal	C1		--	--	--	--	--	--	--	--	--
	C2	C1-2	air	yes-D	no	d and v	d and v	--	--	--	--
	C3	C2-3	air	yes-D	yes-l	d and v	d and v	--	--	--	--
	C4	C3-4	air	yes-D	yes -D	d and v	d and v	yes	Yes	--	--
	C5	C4-5	air	yes-D	yes-l	d and v	d and v	yes	Yes	no	yes
	C6	C5-6	air	yes-l	yes -D	d and v	d and v	yes	Yes	no	yes
	C7	C6-7	air	yes-D	yes-l	d and v	d and v-f	--	Yes	no	yes
	T1	C7-T1	air	yes-l	No	d and v-f	d and v-f	--	Yes	no	--
	T2	T1-2	air	yes-D	Yes	d and v-f	d and v-f	--	--	--	--
	T3	T2-3	air	No	No	no	no	--	--	--	--
	T4	T3-4	air	No	No	no	no	--	--	--	--
	T5	T4-5	no	No	No	no	no	--	--	--	--
Sagittal	C1		--	No	No	--	--	--	--	--	--
	C2	C1-2	air	yes-D	No	d and v	d and v	--	--	--	--
	C3	C2-3	air	yes-D	yes-l	d and v	d and v	--	--	--	--
	C4	C3-4	air	yes-D	yes -D	d and v	d and v	--	--	--	--
	C5	C4-5	air	yes-D	yes-l	d and v	d and v	--	--	no	yes
	C6	C5-6	air	yes-l	yes -D	d and v	d and v	--	--	no	yes
	C7	C6-7	air	yes-D	yes-l	d and v	d and v-f	--	--	no	yes
	T1	C7-T1	air	yes-l	No	d and v-f	d and v-f	--	--	no	--
	T2	T1-2	air	yes-D	Yes	d and v-f	d and v-f	--	--	--	--
	T3	T2-3	air	No	No	no	no	--	--	--	--
	T4	T3-4	air	No	No	no	no	--	--	--	--
	T5	T4-5	no	No	No	no	no	--	--	--	--

ASM= anterior scalene muscle; BP= Brachial plexus; D = dark; d and v= dorsal and ventral nerve root; f= faint; l= light; nf= neural foramen.

**Figure 2:**
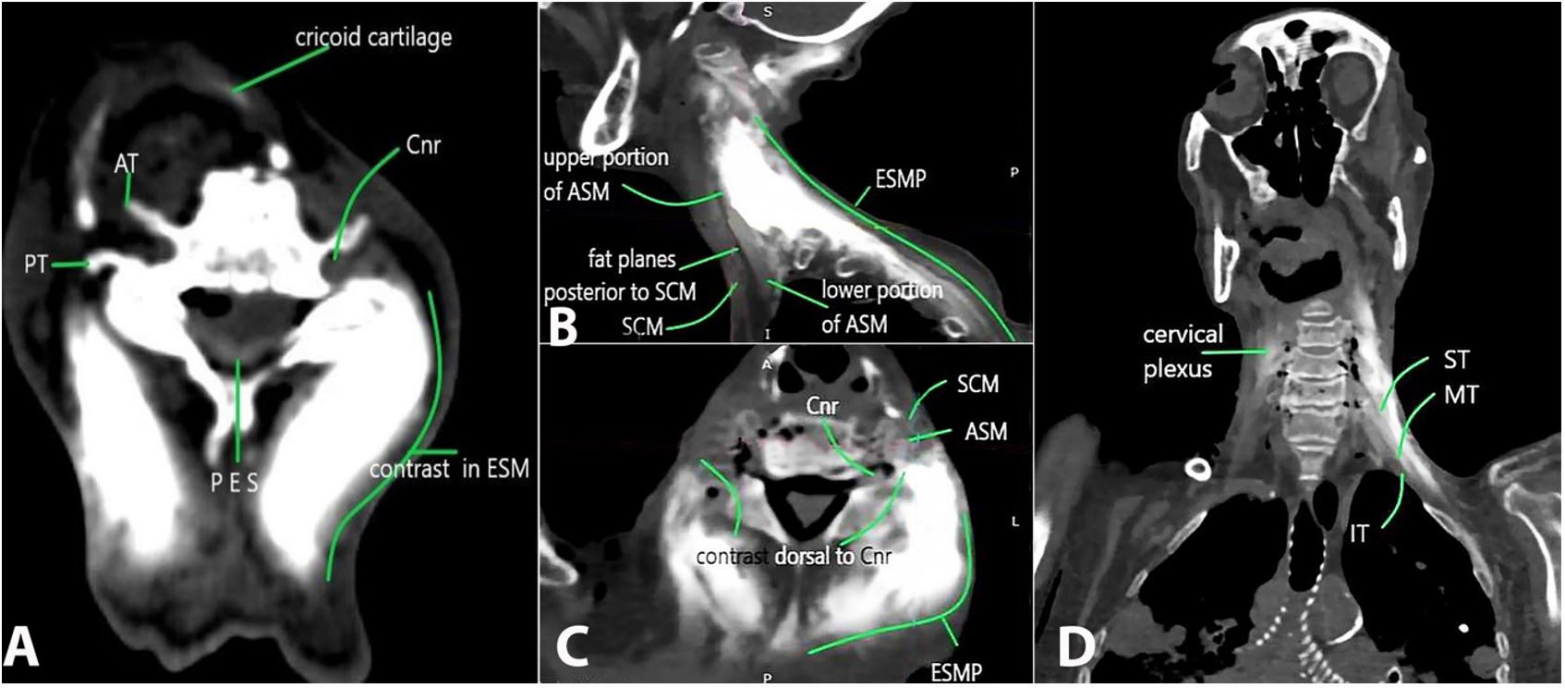
**A** – Axial view demonstrates the posterior epidural spread (PES) (PT-posterior tubercle, AT-anterior tubercle, Cnr- cervical nerve root, ESM-erector spinae muscle); **B** – In the sagittal plane, the contrast delineates the ESMP. The contrast highlights the upper to the mid portion of ASM and the SCM. The fat plane is free of contrast; **C** – In the coronal plane, the contrast delineates bilateral Cnr at level 2, and is dorsal to Cnr 6 on left side. A bilateral PVS spread is seen from C2-6 (SCM -sternocleidomastoid, ASM-anterior scalene muscle, MSM -middle scalene muscle, PSM -posterior scalene muscle; ESM -erector spinae muscle; Cnr -cervical nerve root); **D** – The left superior, middle and inferior trunks are delineated. On the right side a high cervical plexus is observed (ESMP-erector spinae muscle plane, ASM-anterior scalene muscle, MSM-middle scalene Muscle, ST- superior trunk, MT-middle trunk, IT-Inferior trunk).

In cadaver 1, an epidural spread at the C5-6 and C6-7 was visualized in coronal and axial planes bilaterally (Figure 2A). In cadaver 2 in the sagittal section, the contrast delineated the ESM from the level of occiput up to T3-4 (Figure 2B). Contrast spread delineated the upper to mid portion of ASM and SCM muscle, but the fat plane beneath the SCM muscle was free of contrast (Figure 2B). The contrast from the ESP coursed from dorsal to ventral and engulfed the ASM and was visualized at SCM muscle (Figure 2B). In the axial view (Figure 2B), the spread from the left ESP was visualized at the superior surface of ASM and in the fat plane between the ASM and the SCM. In the coronal view (Figure 2C), an extensive bilateral cervico-brachial-thoracic spread was seen. The left superior, middle, and inferior brachial trunks are delineated (Figure 2D). On the right side, the contrast is observed in the cervical plexus (Figure 2D).

### Cadaveric cross-section

**Figure 3:**
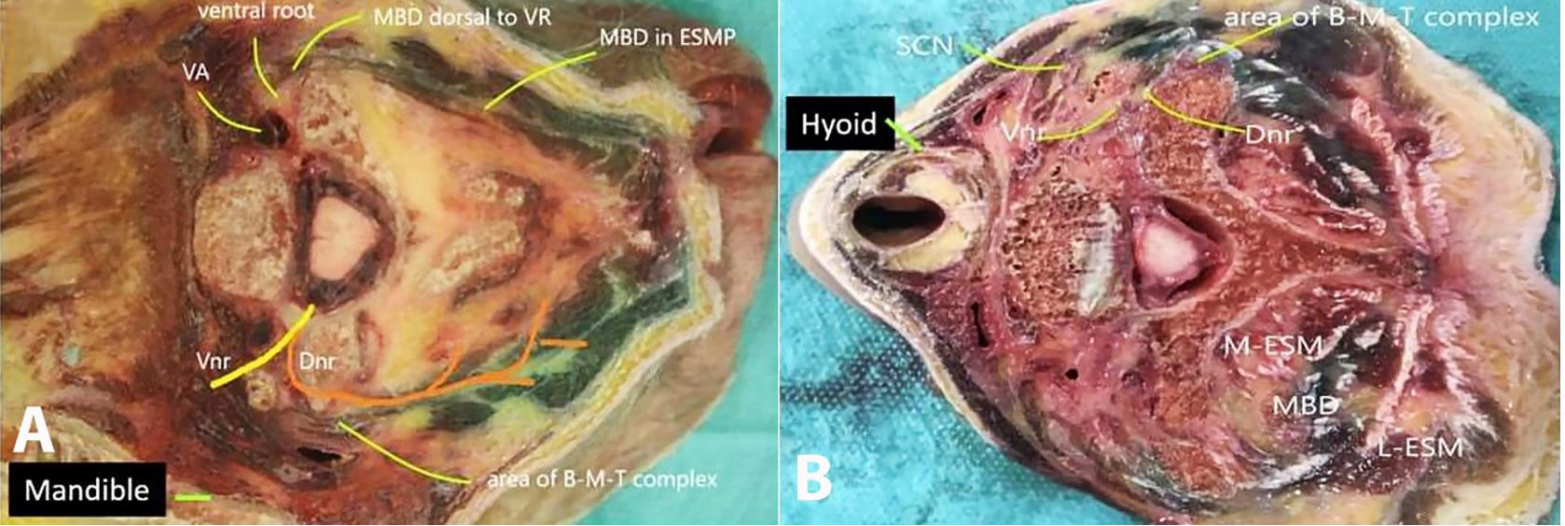
**A** – At the level of mandible - The MBD is seen bilaterally between the lateral and medial erector spinae group of muscles. The MBD extends up to the dorsal of the ventral root emergence on the specimen’s right. The medial limit is close to the facet joint. On the left of the specimen, the spread is dorsal to the TP; **B** – At the level of hyoid – On the right of the specimen, the MBD reaches as far as the ventral rami and close the below the SCM. On the left of the specimen, the MBD courses between the medial and lateral ESM’s. The further course is restricted by the area occupied by the bone-muscle-tendon complex (B-M-T complex). AT- anterior tubercle, TP- transverse process, Vnr-Ventral nerve root, Dnr- dorsal nerve root, PT of TP- posterior tubercle of transverse process, VA- vertebral artery, Cnr- cervical nerve root, B-M-T: bone-muscle-tendon, ESMP- erector spinae muscle plane, MBD- methylene blue dye.

**Figure 4:**
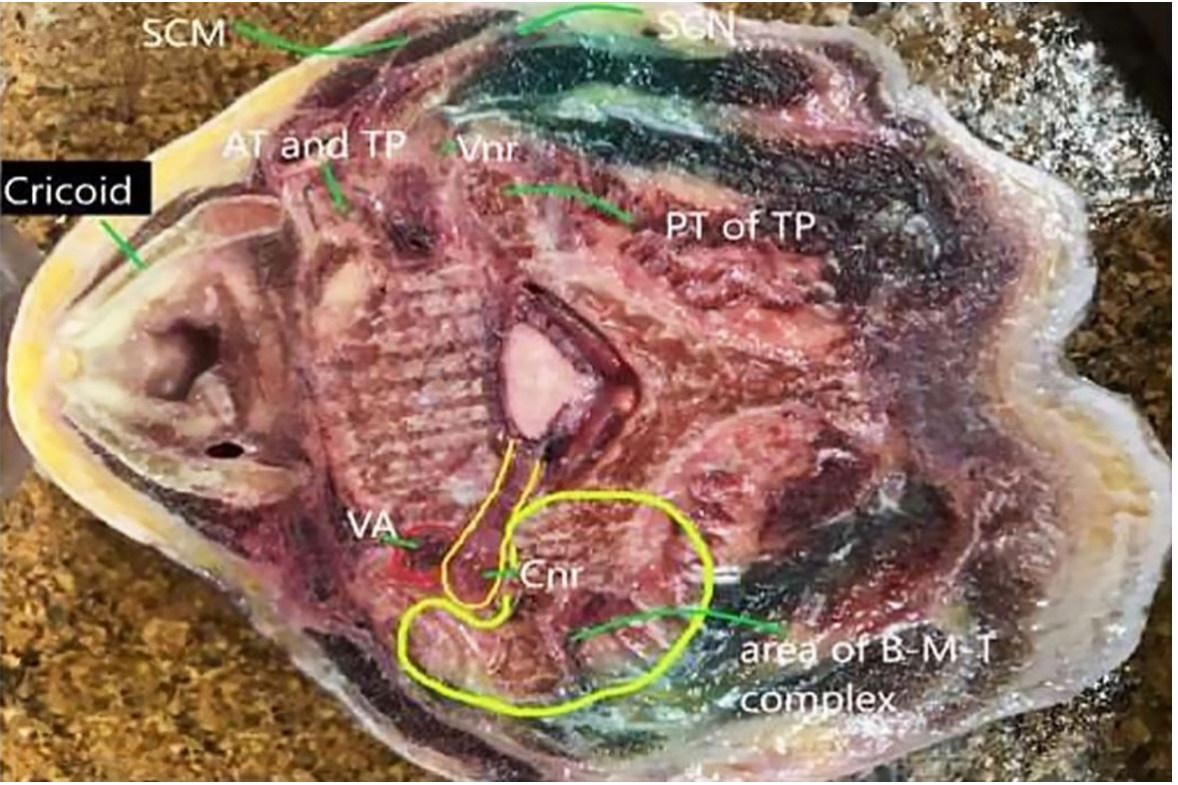
At the level of the cricoid cartilage – On the right of the specimen the MBD reaches as far as the dorsal of the ventral root and beneath the SCM. On the left of the specimen, the B-M-T complex (delineated by light green) restricts the ventral spread of the dye. AT- anterior tubercle, TP- transverse process, Vnr-Ventral nerve root, PT of TP- posterior tubercle of transverse process, VA- vertebral artery, Cnr- cervical nerve root, B-M-T: bone-muscle-tendon.

The cadaveric cross-sections performed at the level of the angle of the mandible (C2), at hyoid (C3), and the cricoid, i.e., C6 (Figure 3A, 3B and Figure 4) were grossly examined. A spread was observed dorsal to the ventral rami in all specimens at the level of the cricoid cartilage as the contrast traveled between the lateral and medial erector spinae group of muscles. A lateral spread was visible posterior to the SCM. No epidural spread was visible. No spread was visible at the extraneural foramina (beyond the tubercles along the nerve sheath), in the neural foramina (between the anterior and posterior tubercle), and at the ASM area.

## DISCUSSION

We observed in fresh human cadaver, using CT imaging that injections deep to the ESP at the level of thoracic CTJ 1 with the needle bevel introduced from caudal to cephalad produces a distribution of the volume in ESP from T5-6 to C1-occiput. CT contrast images of both cadavers depicted spread in the extraforaminal and foraminal levels from C4- T1. Cadaver 1 demonstrated an epidural spread at C5-6 and C6-7 in the axial and coronal planes. Cadaver 2, in the axial and sagittal planes, exhibited contrast spread in the superior surface of ASM and the fat planes beneath the SCM. The cadaveric cross-sections revealed MBD along the entire course of the dorsal rami, sandwiched between the medial and lateral erector spinae group of muscles. A faint tinge of dye appeared on to the dorsal area of the ventral root. There was no dye stain close to the anterior tubercle and pre-tracheal fascia. MBD was not visualized in the epidural space.

Emerging at the intervertebral foramen the cervical nerve trunk divides into a ventral ramus and a dorsal ramus. The dorsal rami turn around the facet joint posteriorly, innervating it with an articular branch. The dorsal rami descend dorsally between the lateral (splenius capitis and semispinalis capitis) and medial (semispinalis cervicis and multifidus) ESM innervating them, the ligaments and terminates in a cutaneous branch. ^10^ The ventral rami descend anterolaterally through the scalene muscles to form the brachial plexus. The dorsal rami emerge through the anatomical tunnel formed by the posterior tubercle and the splenius capitis. At its emergence, the muscle attachments at the posterior tubercle and facet joint forms an important barrier, which should probably inhibit local anesthetic diffusion from the dorsal to the ventral aspect of the posterior tubercle. In spite of this barrier, injections at thoracic CTJ 1 found its path towards the brachial plexus, extraforaminal and neural foraminal areas, and the epidural space (Figure 222C).

With its consistent spread along dorsal rami, the cervical ESPB would benefit surgical procedures on the dorsal side in acute and chronic pain conditions affecting the neck and upper back.^8^^,^^9^ A cadaveric study using injections of MBD dorsal to C6 TP demonstrated consistent stain of ventral nerve roots in all specimens and phrenic nerve in one specimen.^11^ The study involved injection and dissection in a prone position and then repositioned to supine followed by anterior dissection. This could lead to the spread of dye to unwanted areas affecting the analysis. In our study, we used a combination of radiocontrast and MBD in 2 cadavers followed by CT scan and then cross-sectional studies in the prone position. By doing this, unnecessary change of position was avoided. We observed extraforaminal and foraminal diffusion in all 4 specimens. The spread at C5-6 and C6-7 in posterior epidural space was observed in cadaver 1 in axial view (Figure 2A) and to the ASM in cadaver 2 on the right (axial and coronal view, Figure 2B).

We agree that the sample size was a limitation. Moreover, results of cadaveric contrast spread may not be extrapolated to clinical cases due to several factors. The air in the neuraxial space was a major limiting factor in cadaver 2 to assess epidural spread. A pre-study CT scan of the cadavers would exclude several unwanted elements like air pockets and cervical scoliosis in areas of interest. MBD solutions have an unrestricted flow against the highly viscous latex injections, which solidifies immediately. Probably a combination of latex and MBD in future studies would enable us to understand the spread better. In the future, similar cadaveric injections followed by dissection in prone position will establish and solidify the concept of limited ventral spread from injection dorsal to first thoracic CTJ.

## CONCLUSION

We conclude that the mechanism of analgesic effect of the cervical ESPB injected at the level of the T1 from caudal to cephalad could be through its action on the dorsal rami, via dorsal spread on the ventral root and occasional extraforaminal/ neural spread of cervical nerves.

The results of our study, which is an anatomic and radiological correlation of the spread of dye, needs to be investigated clinically.
